# Drought-induced microbial dynamics in cowpea rhizosphere: Exploring bacterial diversity and bioinoculant prospects

**DOI:** 10.1371/journal.pone.0320197

**Published:** 2025-03-25

**Authors:** Boshra Ahmed Halo, Yaqeen A. S. Aljabri, Mahmoud W. Yaish

**Affiliations:** Department of Biology, College of Sciences, Sultan Qaboos University, Muscat, Oman; University of Salento: Universita del Salento, Italy

## Abstract

Rhizospheric bacterial communities in plants contribute to drought resilience by promoting plant-soil interactions, yet their biodiversity and ecological impacts are not fully characterized. In cowpeas, these interactions may be crucial in enhancing tolerance to drought conditions. In this study, cowpea plants were subjected to drought treatment, the soil attached to the roots was collected, environmental DNA (e-DNA) was extracted, and the bacterial communities were identified as amplicon sequence variants (ASVs) by metagenomics analysis of the 16S rRNA gene. Microbial communities under drought and control conditions were analyzed using taxonomy and diversity metrics. The sequencing results revealed 5,571 ASVs, and taxonomic analysis identified 1,752 bacterial species. Alpha and beta diversity analyses showed less conserved microbial community structures and compositions among the samples isolated from the rhizosphere under drought conditions compared to untreated samples, implying the enhancement effect of drought on species’ biodiversity and richness. The differential accumulation analysis of the bacterial community identified 75 species that accumulated significantly (*P* ≤  0.05) in response to drought, including 13 species exclusively present, seven absent, and 46 forming a high-abundance cluster within the hierarchical heatmap. These species were also grouped into specific clades in the phylogenetic tree, suggesting common genetic ancestry and potentially shared traits associated with drought tolerance. The differentially accumulated bacterial list included previously characterized species from drought and saline habitats. These findings suggest that drought stress significantly alters the composition and abundance of epiphytic bacterial communities, potentially impacting the rhizosphere’s ecological balance and interactions with cowpeas. The results highlight microbial adaptations that enhance plant resilience through improved stress mitigation, providing meaningful understandings for advancing sustainable agriculture and developing microbial-based strategies to boost crop productivity in drought-prone regions.

## Introduction

Drought is a major environmental stress that disrupts plant growth, reduces photosynthesis, and significantly limits the productivity of certain crops [1]. Globally, yield losses of important crops were reported in many countries due to drought impacts. For example, drought due to global warming has significantly reduced maize production in northeast China over the past five decades [2], soybean production in the United States [3], and crop yields in Australia, where drought spells have reduced yields by more than 50% [4]. In the Middle East, several reports documented extraordinary temperature increases over the last few decades, which were associated with drought due to the reduction in rainfall totals over the past decades, as revealed in many assessments in this region [5,6].

Cowpea (*Vigna unguiculata* L. Walp.) is a nutrient-dense legume, rich in protein and carbohydrates, low in fat, and featuring a complementary amino acid profile that pairs well with cereal grains, making it a valuable meat substitute, particularly in developing countries [7]. In addition to its nutritional benefits, cowpea exhibits resilience to abiotic stresses such as drought, heat, and salinity, which has led to its widespread use in cropping systems in arid and semiarid regions [8,9].

Rhizospheric bacterial communities in plants contribute to drought resilience by enhancing soil affordability and plant viability, yet their biodiversity and ecological impacts are not fully characterized [10]. Harnessing growth-promoting bacteria offers a promising approach to mitigating the effects of drought on plant growth and productivity [11]. Bacteria naturally associated with plant roots are an ideal source for such treatments, as they do not disrupt the microbial balance in the soil or pollute soil, plants, and groundwater, unlike synthetic fertilizers [12]. Therefore, native rhizospheric bacteria were previously identified, isolated, and tested for their ability to enhance plant growth under drought conditions, and then they were used to develop biofertilizers [13]. Since cowpeas are relatively drought-tolerant, it is critical to identify root-associated bacterial communities and evaluate their enrichment dynamics in response to drought conditions for potential future isolation and utilization as inocula to protect plants against drought.

The effect of specific abiotic stresses, such as salinity, on the rhizosphere microbiome of cowpeas has been explored in previous studies. For example, Mukhtar, Hirsch [14] analyzed the distinct rhizosphere and nodule microbiomes in salinity-stressed cowpea soils, identifying bacterial isolates with plant-growth-promoting traits and emphasizing their potential as future crop inoculants. Additionally, Dubey, Bhattacharjee [15] analyzed microbiome-mediated rhizosphere engineering in *Vigna radiata*, showing fungal community shifts through internal transcribed spacer (ITS)-based sequencing that correlated with improved plant growth and salinity stress mitigation. However, no available study has yet investigated the impact of drought stress on the rhizosphere microbial community using high-resolution microbial profiling methods that identify unique e-DNA sequences at single-nucleotide resolution, such as ASVs, which leaves a critical knowledge gap in understanding drought-specific microbial adaptations.

Given that most bacterial species are non-culturable without prior knowledge of their characteristics, the novel use of next-generation sequencing (NGS) offers an exceptional opportunity to identify drought-adapted epiphytic and rhizospheric bacteriome associated with cowpeas, which enable the precise detection of specific rhizobacteria under drought conditions while overcoming the limitations of traditional cultivation methods. This analysis can provide detailed information about the epiphytic rhizobacterial communities in plants, including their diversity, taxonomic composition, and relative abundance when grown under drought [16,17]. Additionally, it can help identify key rhizobacteria that play a critical role in enhancing cowpeas’ resilience to drought conditions.

This report aims to characterize the epiphytic rhizobacterial community in cowpeas, study their biodiversity response to drought, and identify key bacterial species of potential adaptation to water scarcity and have plant growth-promoting capacity. To achieve these goals, cowpea plants were grown under optimal (control) and drought conditions, e-DNA samples were extracted from their rhizospheres, and epiphytic bacterial amplicon sequence variants (ASVs) and their accumulation patterns were determined using 16S rRNA gene analysis and NGS approaches. The results showed that ASVs of high diversity led to substantial shifts in the bacterial community in response to drought stress in the cowpea rhizosphere.

## Materials and methods

### Plant growth conditions and drought treatment

Cowpea (*Vigna unguiculata* subsp. *unguiculata*) seeds were surface sterilized by soaking in 70% ethanol for 3 minutes, followed by immersion in a 10% bleach solution (5% sodium hypochlorite) for 40 seconds with gentle swirling. The seeds were rinsed thoroughly three times with sterilized water. Eight pots were filled with soil from Al Seeb area, Muscat, Oman (23.6472° N, 58.1456° E). Four seeds were planted in each pot and then covered with a thin layer of soil. The pots were placed in a growth room set at 24°C with a 16-hour light/8-hour dark cycle and watered every 2 to 3 days for six weeks. After six weeks, the pots were divided into two groups of eight. Watering continued for one group (control), while the second group was subjected to drought stress by withholding water for three consecutive weeks (drought). Nine weeks after planting, the roots were carefully removed from four regularly watered pots and four drought-treated pots. Moisture and electrical conductivity (EC) were measured using ECH2o software (www.metergroup.com) connected to a data logger and a 5TE sensor (METER, Pullman, USA). The 5TE sensor was placed inside the pots, close to the plants’ roots, while taking measurements. The soil pH was measured using Hanna, HI2550 model pH meter (HANNA Instruments Inc., USA), as previously described [18]. The total organic carbon (TOC) was measured based on the method previously described by Walkley and Black [19], and the CHNS/O elements of the soil were measured using an automatic CHNS/O analyzer (PerkinElmer Series II CHNS/O, USA), as previously described [20].

### Measurement of plant growth parameters

The length of each plant stem was recorded using a measuring tape. The pots were cautiously emptied to prevent damage to the delicate roots, and the shoots were separated from the roots with scissors. Fresh shoot weights were then determined using an electrical scientific scale (Highland (HCB302), Adam Equipment, UK). The dry weight of the shoots and root was measured after being air-dried for three weeks, incorporating a minor modification to the previously established procedure [21].

### Rhizospheric soil sample collection and metagenomic analysis

The epiphytic bacterial communities associated with the roots were studied by collecting the most proximate soil to the cowpea roots. The roots were gently shaken to remove large soil clumps, and the soil particles closely adhering to the roots were collected by brushing. The e-DNA was extracted from the soil samples using DNeasy PowerBiofilm kit (Cat. number 24000-50) (Qiagene, Hilden, Germany) following the manufacturer’s instructions. This DNA extraction kit was selected due to its high efficiency in extracting sufficient DNA from the soil for the analysis. The e-DNA samples’ concentrations and purity were determined using a Nanodrop2000 spectrophotometer (Thermo Scientific™, USA) and run on 1% TAE gel electrophoresis. All e-DNA samples were used to identify the bacterial community through 16S rRNA gene metagenomic amplicon sequencing, using high-throughput sequencing that targeted the gene (V3-V4) region. This region of the 16S rRNA gene was targeted due to its high taxonomic resolution and broad applicability in microbial community profiling. The oligonucleotides Bakt_341F (5’-CCTACGGGNGGCWGCAG-3’) and Bakt_805R (5’-GACTACHVGGGTATCTAATCC-3’) were employed, and the DNA sequencing was performed on an Illumina MiSeq system with 300 bp paired-end reads. DNA sequencing was outsourced as a service provider to Macrogen, South Korea. Eight libraries were prepared, four from soil attached to roots of plants grown under optimum conditions (control group) and four from cowpea plants grown under drought conditions (drought group). The eight libraries were prepared with the Herculase II Fusion DNA Polymerase and Nextera XT Index V2 Kit, following the 16S rRNA metagenomic sequencing library preparation protocol for the Illumina MiSeq System (Part no. 15044223 Rev B. Illumina, San Diego, CA, USA).

### Computational data analysis

Initially, Cutadapt was used to remove adapter sequences for the DNA raw data trimming. DADA2 (v1.18.0) [22] was employed for ASV generation, which included quality filtering, error correction, and chimera removal. Sequence length filtering of 16S rRNA sequences was performed in R to retain biologically relevant reads.

Taxonomic identification of ASVs was performed using BLAST2 + (v2.9.0) against a curated 16S rRNA database (version NCBI_16S_20231219), while QIIME (v1.9) [23] was used exclusively to calculate alpha diversity metrics, including the Shannon index, Gini-Simpson index, and PD whole tree, to evaluate species complexity within individual samples and sample groups. The number of observed species (ASVs) was also reported.

Quality control was performed using DADA2 and QIIME, which provide filtering, trimming, taxonomic assignment, and diversity analysis. Beta diversity analysis was conducted to illustrate the variations among bacterial community groups (control and drought), excluding the unclassified ASVs, in response to drought treatment using the principal coordinates analysis (PCoA) method and the Morisita similarity index implemented within the Past 3.0 software package [24]. The phylogenetic tree was constructed with a bootstrap test (100 replicates) using the maximum likelihood Tamura-Nei method via Mega software package 11 [25]. The 16S rRNA sequences of species that differentially accumulate at significant (*P* ≤  0.05) levels in response to drought were selected for phylogenetic analysis using the corresponding single ASV of the highest abundance. The counts of different ASVs were summed for each assigned species and utilized in a differential accumulation study based on the four replicates of each treatment. The heatmap complete linkage hierarchical clustering was created to visualize bacterial abundance profiles across different treatments of the differentially accumulated species using PermutMatrix software version 1.9.3 [26]. The data underwent additional analysis for bacterial identification through pairwise comparisons to determine significant differences between groups, utilizing the Post Hoc Tukey Honest Significant Difference (HSD) test in SPSS software version 21.0 (IBM, 2012) with a significance level of *P* ≤  0.05.

## Results

### Drought significantly impacts cowpeas’ growth

The soil water content resulting from the treatment and some growth parameters in cowpea plants were measured to investigate the effect of drought on plant growth and development. The soil moisture measurements around the roots at the end of the experiment for the control and drought-treated plants showed that drought treatment significantly (*P* ≤  0.05) reduced soil moisture. While the average soil moisture in the control group was 0.188 ± SD = 0.003 m3/m3, the drought-treated plants’ soil moisture group dropped to 0.058 ± SD = 0.005 m3/m3. The E.C. of the control group exhibited a conductivity of 0.38 ±  SD =  0.0264 mS/cm, however, the E.C. was not detectable in the drought group using the 5TE sensor. The pH value of the soil used in this experiment was 8.05, and the total organic carbon (TOC) percentage was 0.51%, indicating an alkaline soil with low organic carbon contents. The soil analysis also showed that it contained 5.53% carbon (C), 0.66% hydrogen (H), and 0.02% sulfur (S) by total composition, implying that most of the carbon in the soil is inorganic.

**Fig 1:**
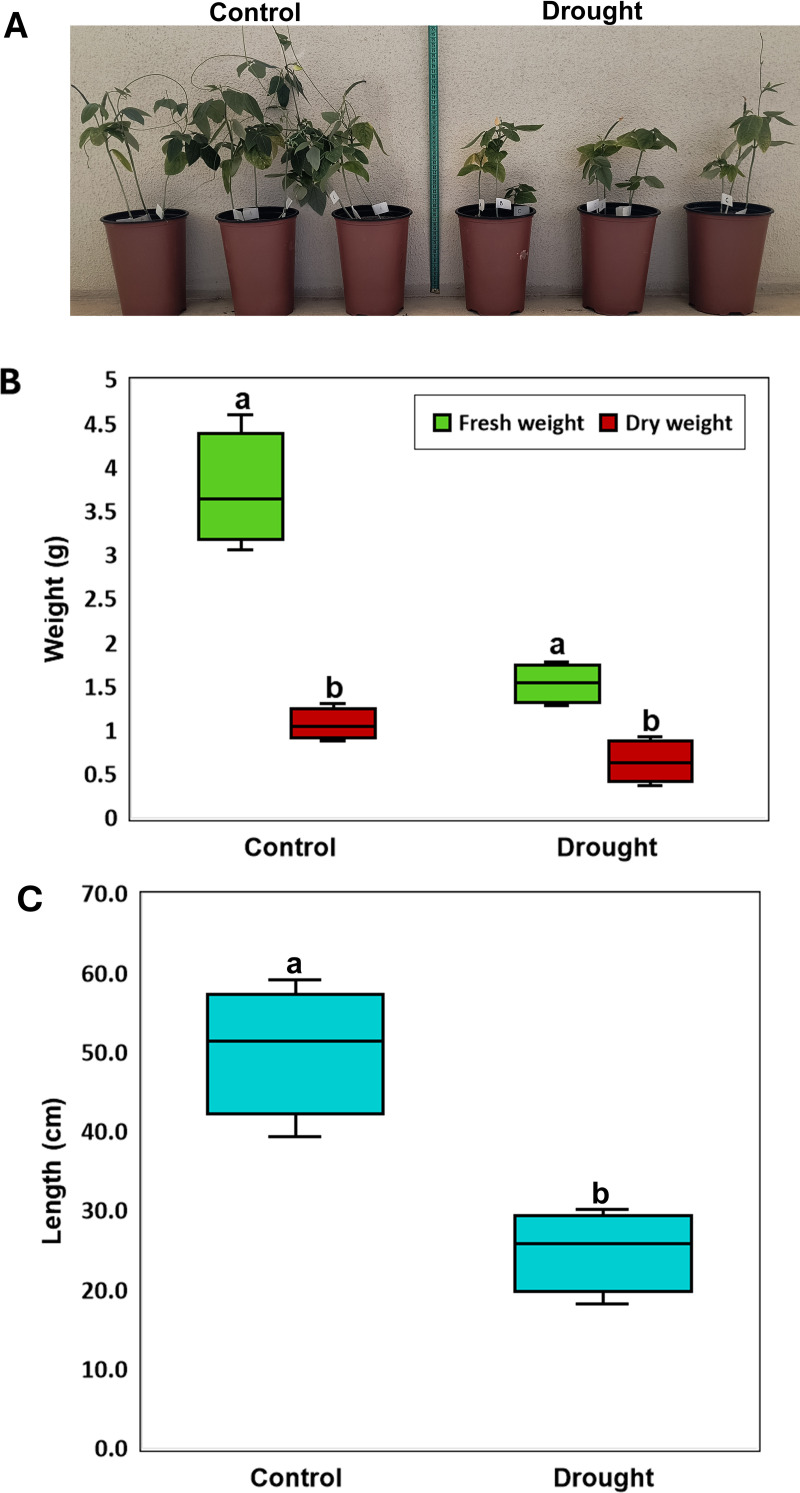
The drought treatment remarkably affects the growth of cowpeas, as evidenced by the overall structure compared to the untreated plants (control) (A). Drought significantly impacts shoot fresh and dry weights (B), and the stem lengths of the plants (C). The letters above the box plots indicate a significant value of *P* ≤  0.05.

The plant growth was strongly impacted by drought, as shown by the plants’ stunt apparent due to growth inhibition (Fig 1A), and the reduction of the measured plant growth parameters compared to plants that grew under control conditions, including the fresh shoot weight of (3.7 ± SD =  0.6 vs. 1.5 ± SD = 0.2 g), the dry weight (1.1 ± SD =  0.2 vs. 0.6 ± SD =  0.2 g), and the stem length (50.3 ± SD = 8.2 vs. 25 ± SD =  5.1 cm) of cowpea plants. These results represent a significant (*P* ≤  0.05) reduction in the fresh shoot weight by approximately 60%, dry weight by 45% (Fig 1B), and total stem length by about 50% (Fig 1C). The root dry and fresh weights were measured; however, only the root fresh weight showed a significant difference in response to drought treatment (*P* ≤  0.05). The average root fresh weights were 0.64 g (SD ±  0.2) for the control and 0.35 g (SD ±  0.1) for the drought-treated plants, indicating a significant reduction due to drought.

### High-throughput sequencing of the 16S rRNA gene and ASV identification

High-throughput sequencing of the 16S rRNA gene libraries generated a large volume of raw sequence data. The sequencing of the eight libraries resulted in 943,441,758 pb with an average of 58,965,110 bp per sample. Initially, the sequencing process produced 833,299 raw reads, which were then subjected to various filtration processes, and the data was refined to ensure high-quality reads for downstream analysis. The library sequence produced an average of ~ 208 K reads per library. The libraries’ quality scores (Q30/Q20 ratio) range between 0.89 and 0.91, indicating high sequence quality. After adapter and primer trimming, length trimming, quality filtering, and ASV length filtration, a total of 458,525 reads remained. The sequencing results revealed 5,571 ASVs, including 887 unclassified variants (S1 Table). These unclassified ASVs often lack sufficient taxonomic resolution and may represent low-abundance/quality or ambiguous sequences, which could introduce noise and obscure critical patterns in microbial dynamics, therefore they were excluded from further analysis in this study. The length of the ASVs ranged from 402 to 428 bp, with an average of 415 bp. The taxonomic annotation of the ASV classified them into 30 phyla, 75 classes, 162 orders, 328 families, 909 genera, and 1,751 species, accounting for 323,446 abundance values. The taxonomic classification of ASVs across the eight libraries revealed that the phylum Pseudomonadota was the most abundant, with the Alphaproteobacteria class being the most dominant. The order Hyphomicrobiales showed the highest prevalence, while the family Xanthomonadaceae stood out as the most prominent. At the genus level, Nocardioides was the most abundant, and the species *Actinomarinicola tropica* had the highest representation.

### Drought enhanced higher species richness and evenness in the epiphytic bacterial communities

**Fig 2:**
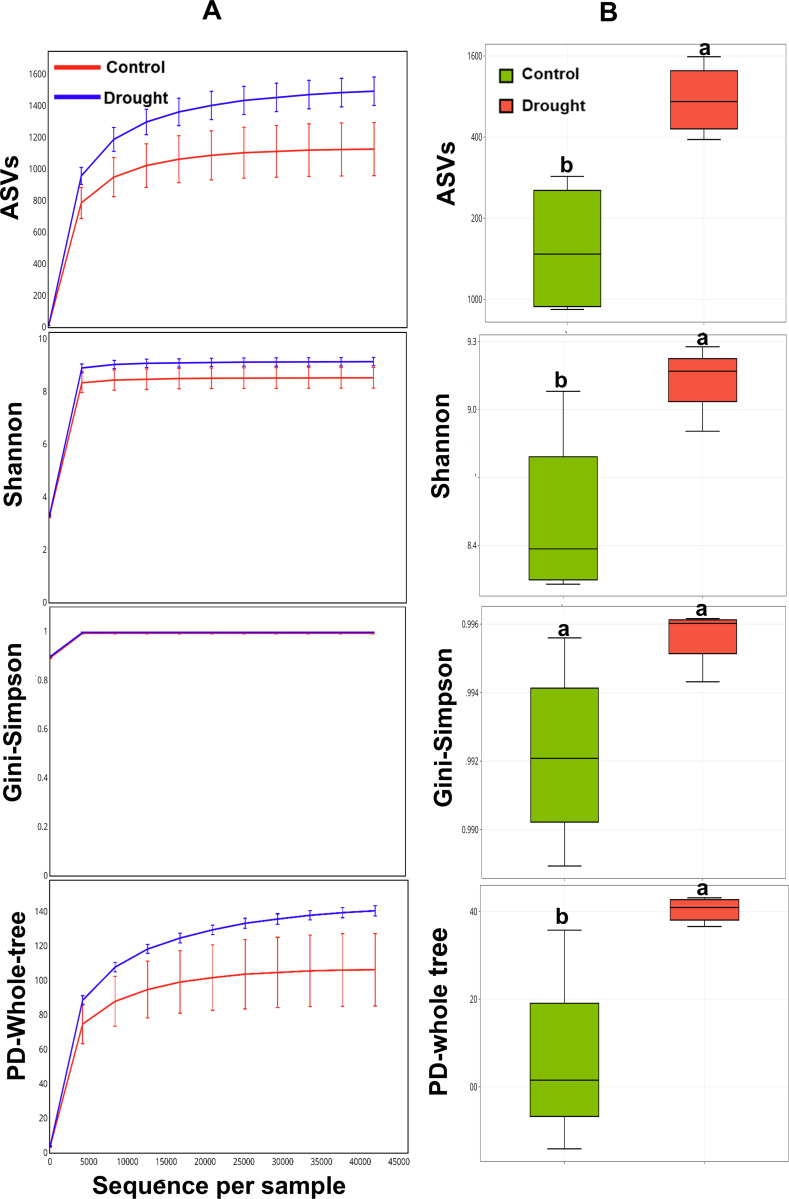
Alpha diversity analysis of epiphytic rhizobacteria associated with cowpea roots under control and drought conditions. Rarefaction curve analysis illustrating the sequencing depth of ASVs in bacterial communities from both treatments, assessed using various diversity indices (A). Box plots display the distribution of alpha diversity across the groups, with diversity measured by multiple indices (B). Different letters above the box plots indicate significant levels (*P* ≤  0.05).

Alpha diversity analysis was used to assess microbial diversity in rhizosphere soil samples from control and drought treatments using the rarefaction-based and overall alpha biodiversity methods. The rarefaction curves were constructed based on the number of sequences for both control and drought-treated groups, providing comprehension into diversity within the epiphytic communities in the rhizosphere of cowpea plants (Fig 2A).

This approach ensures fair comparisons across samples with varying sequencing depths, offering a balanced evaluation of microbial richness and evenness under different conditions. The comparative analysis of the ASV index between community sample groups subjected to drought and those in control treatment demonstrated higher values of biodiversity index under drought. Similarly, the Shannon index’s comparative analysis revealed that ASVs identified under drought treatment showed higher microbial diversity and evenness than those under control treatment, indicating a more significant number of unique taxa. However, the Gini-Simpson index-based rarefaction curves demonstrated that both groups exhibited similar levels of species dominance, with comparable abundance of the most common species. Phylogenetic diversity (PD-Whole Tree) analysis indicated that microbial communities under drought treatment had greater phylogenetic diversity than those in control treatment, reflecting a broader range of evolutionarily distinct taxa. The alpha diversity analysis was also performed to assess the overall microbial diversity among community groups, irrespective of sequencing depth. The results confirmed that the microbial communities in cowpea plants subjected to drought exhibited higher diversity across most indices (Fig 2B). For instance, drought treatment significantly (*P* ≤  0.05) increased in ASVs, suggesting a higher richness of unique taxa than the control.

### Metagenomic analysis revealed higher diversity among communities in response to drought

**Fig 3:**
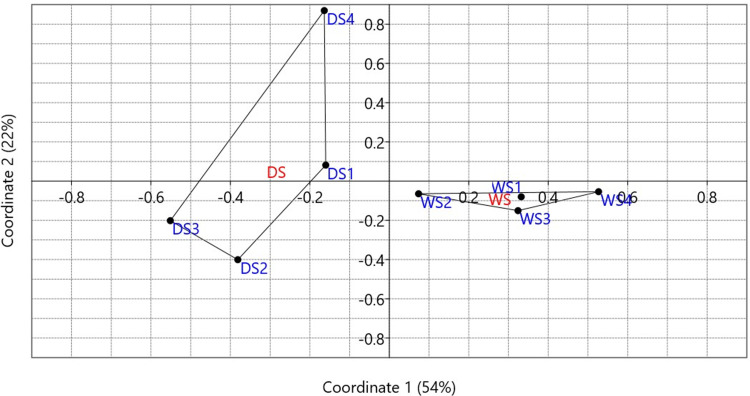
Beta diversity analysis of the identified bacterial species from four independent epiphytic rhizobacterial communities of cowpeas grown under control (WS1-WS4) and drought (DS1-DS4) conditions, based on species abundance. Principal coordinates analysis (PCoA) illustrates 54% and 22% of the total variation explained by the first and second coordinates, respectively.

Beta diversity analysis was used to visualize the overall differences in bacterial communities in rhizospheric soil samples isolated from plants grown under control and drought conditions and assess the consistency of replicates. The diversity analysis was carried out using the principal coordinates analysis (PCoA) of the species abundance data of the eight replicates. The results showed that the PCoA analysis explained 76% of the total variation within the samples, with the first and second coordinates accounting for 54% and 22%, respectively. The results also showed that bacterial communities from plants grown under control and drought conditions are well-separated on the ordination plot. The bacterial communities of plants grown under the control condition are more tightly clustered, whereas those of drought-treated plants showed more variation among the bacterial community replicates (Fig 3).

### Metagenomic analysis revealed differential bacterial abundance due to drought

**Fig 4:**
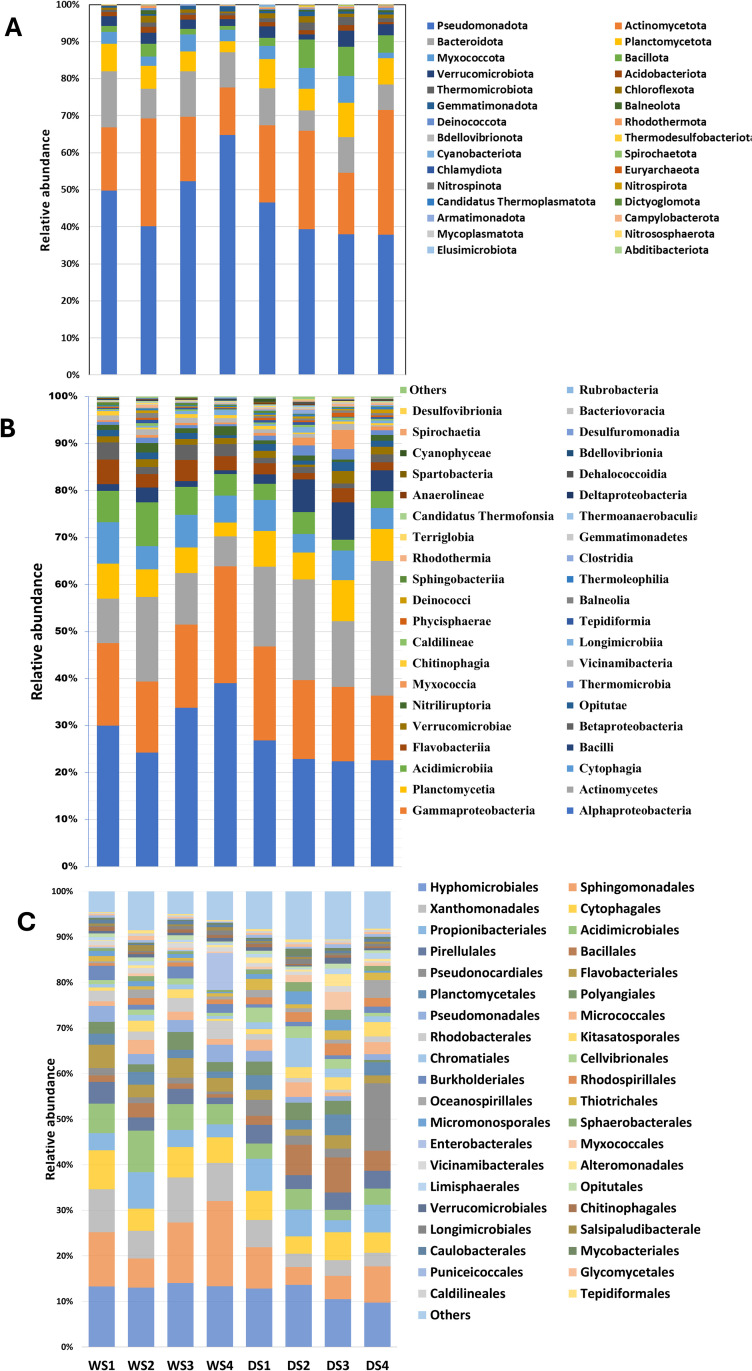
The relative taxonomic abundance of the top taxa units classified at phylum (A), class (B), and order (C) levels of the microbial communities identified when cowpea plants grown under control (WS1-WS4) and drought (DS1-DS4) conditions.

The taxonomic annotation of the ASVs accompanied by statistical analysis based on the relative abundance of each taxonomic unit showed that the phyla Bacillota and Euryarchaeota exhibited a significantly (*P* ≤  0.05) higher abundance in the epiphytic bacterial communities of cowpeas grown under drought treatment than in control (Fig 4A, Fig S1A, S2 Table). The classes Alphaproteobacteria, Bacilli, Acidimicrobiia, Flavobacteriia, Betaproteobacteria, Bacteriovoracia, Halobacteria, and Syntrophobacteria differentially abundant epiphytic bacterial ASVs in cowpeas at a significant (*P* ≤  0.05) level, with Bacilli being the only class showing increased abundance under drought treatment (Fig 4B, S1B Fig, S2 Table). At the order level, Bacillales, Xanthomonadales, Acidimicrobiales, Flavobacteriales, Rhodospirillales, Thiotrichales, and Pseudomonadales were among the significantly (*P* ≤  0.05) differentially abundant epiphytic bacterial ASVs. Notably, Bacillales, Rhodospirillales, and Thiotrichales orders were more abundant under the drought treatment (Fig 4C, S1C Fig, S2 Table).

The taxonomic analysis identified 1,752 bacterial species at the species level with 323,446 abundance values (S3 Table). Statistical analysis showed that 75 bacterial ASVs differentially accumulated at significant (*P* ≤  0.05) levels in the rhizosphere of the cowpeas in response to the drought treatment. Following drought treatment, 13 bacterial species explicitly accumulated in the cowpea epiphytic community, while seven were absent (Table 1).

**Table 1:** Differential accumulation of 75 rhizospheric bacterial species identified in cowpeas under drought treatment, with significant differences at *P* ≤  0.05.

Species	Control	Drought	*P*- value	Species	Control	Drought	*P*- value
*Silicimonas algicola*	85.00	23.25	0.000	*Bremerella alba*	29.50	142.25	0.023
*Nitrobacter winogradskyi*	29.75	0.00	0.000	*Tistlia consotensis*	59.50	176.75	0.023
*Devosia enhydra*	0.00	16.75	0.001	*Lysobacter yangpyeongensis*	119.50	23.00	0.024
*Pelagibacterium halotolerans*	20.50	53.00	0.001	*Maricaulis maris*	24.00	149.75	0.026
*Rhizobium helanshanense*	936.75	320.00	0.001	*Oceanipulchritudo coccoides*	2.75	24.75	0.026
*Croceibacterium salegens*	50.00	0.00	0.001	*Actinomarinicola tropica*	2218.75	1132.00	0.027
*Luteimonas pelagia*	1233.25	299.50	0.002	*Haliea atlantica*	41.75	169.75	0.028
*Labedaea rhizosphaerae*	3.50	25.25	0.002	*Paenibacillus antri*	3.50	20.25	0.029
*Kofleria flava*	87.00	17.25	0.002	*Pseudobacteriovorax antillogorgiicola*	2.50	7.50	0.031
*Mesorhizobium thiogangeticum*	109.50	29.75	0.003	*Kocuria oceani*	0.00	12.75	0.031
*Vulgatibacter incomptus*	0.75	23.75	0.003	*Bacillus seohaeanensis*	0.00	28.25	0.032
*Luteimonas weifangensis*	93.00	10.25	0.003	*Thermochromatium tepidum*	0.00	1.75	0.032
*Imperialibacter roseus*	20.00	65.50	0.004	*Pelagerythrobacter aerophilus*	16.75	65.50	0.032
*Methylophaga thalassica*	0.50	9.25	0.005	*Oxalicibacterium faecigallinarum*	18.50	2.50	0.032
*Sphingomonas changnyeongensis*	16.50	3.00	0.005	*Pseudonocardia saturnea*	0.00	262.00	0.033
*Hyphomicrobium hollandicum*	13.75	2.25	0.006	*Crocinitomix algicola*	5.00	19.00	0.033
*Ornithinicoccus halotolerans*	30.75	124.75	0.007	*Nocardioides marinus*	101.50	289.50	0.033
*Methylophaga thiooxydans*	134.50	571.00	0.007	*Brassicibacter thermophilus*	8.75	0.00	0.033
*Allomuricauda ruestringensis*	0.00	21.25	0.009	*Nordella oligomobilis*	8.75	0.00	0.033
*Devosia geojensis*	59.00	128.75	0.009	*Shinella zoogloeoides*	609.25	230.25	0.033
*Halobacteriovorax litoralis*	0.00	4.00	0.010	*Flavobacterium daejeonense*	39.25	0.00	0.033
*Pirellula staleyi*	63.25	11.00	0.011	*Streptomyces gobiensis*	0.00	11.50	0.034
*Marinobacter bryozoorum*	3.00	10.25	0.012	*Kangiella koreensis*	22.25	78.50	0.034
*Tessaracoccus arenae*	0.00	7.75	0.012	*Adhaeretor mobilis*	22.00	54.75	0.034
*Xanthobacter autotrophicus*	5.50	0.00	0.013	*Pseudooceanicola nanhaiensis*	154.75	61.00	0.034
*Irregularibacter muris*	0.00	7.00	0.014	*Paracoccus pueri*	243.00	42.25	0.036
*Lysobacter rhizophilus*	195.25	47.75	0.014	*Kordiimonas gwangyangensis*	0.00	10.00	0.036
*Solirubrobacter phytolaccae*	22.50	48.50	0.016	*Nocardioides panacisoli*	17.50	88.50	0.039
*Parapontixanthobacter aurantiacus*	24.00	84.50	0.018	*Pseudomethylobacillus aquaticus*	24.50	3.75	0.041
*Nocardioides endophyticus*	33.00	168.50	0.019	*Pseudoxanthomonas kaohsiungensis*	136.25	0.00	0.041
*Qipengyuania mesophila*	255.75	51.25	0.019	*Halobacteriovorax marinus*	0.75	4.50	0.043
*Thermostilla marina*	25.75	80.75	0.020	*Streptomyces sediminis*	10.25	119.00	0.045
*Aciditerrimonas ferrireducens*	106.00	58.25	0.021	*Paracoccus gahaiensis*	77.75	3.75	0.045
*Rubinisphaera italica*	14.50	65.50	0.021	*Nakamurella leprariae*	0.00	11.75	0.047
*Thalassoroseus pseudoceratinae*	22.25	69.50	0.021	*Alishewanella alkalitolerans*	2.25	12.75	0.047
*Methylobacillus flagellatus*	17.50	6.00	0.021	*Halocatena pleomorpha*	3.25	9.00	0.048
*Breoghania corrubedonensis*	0.00	12.50	0.022	*Parafrankia soli*	32.75	3.25	0.050
*Devosia pacifica*	15.75	51.75	0.022				

Among the differentially accumulated ASVs were bacterial species previously identified in drought conditions such as *Methylophaga* spp.*, Nocardioides* spp*., Ornithinicoccus halotolerans, Paenibacillus* spp., and *Pseudonocardia saturnea*, and species previously identified in saline environments such as *Adhaeretor mobilis, Halobacteriovorax* spp.*, Haliea atlantica, Halocatena pleomorpha*, *Kangiella koreensis, Marinobacter bryozoorum, Maricaulis maris, Ornithinicoccus halotolerans, Pelagibacterium halotolerans*, and *Tistlia consotensis.* The species list also includes bacterial species previously identified in desert habitats, such as *Nocardioides spp*., *Pseudonocardia saturnea*, and *Streptomyces sediminis*, and also in the hot spring, such as *Thermochromatium tepidum*.

### Hierarchical clustering of drought-responsive species

**Fig 5:**
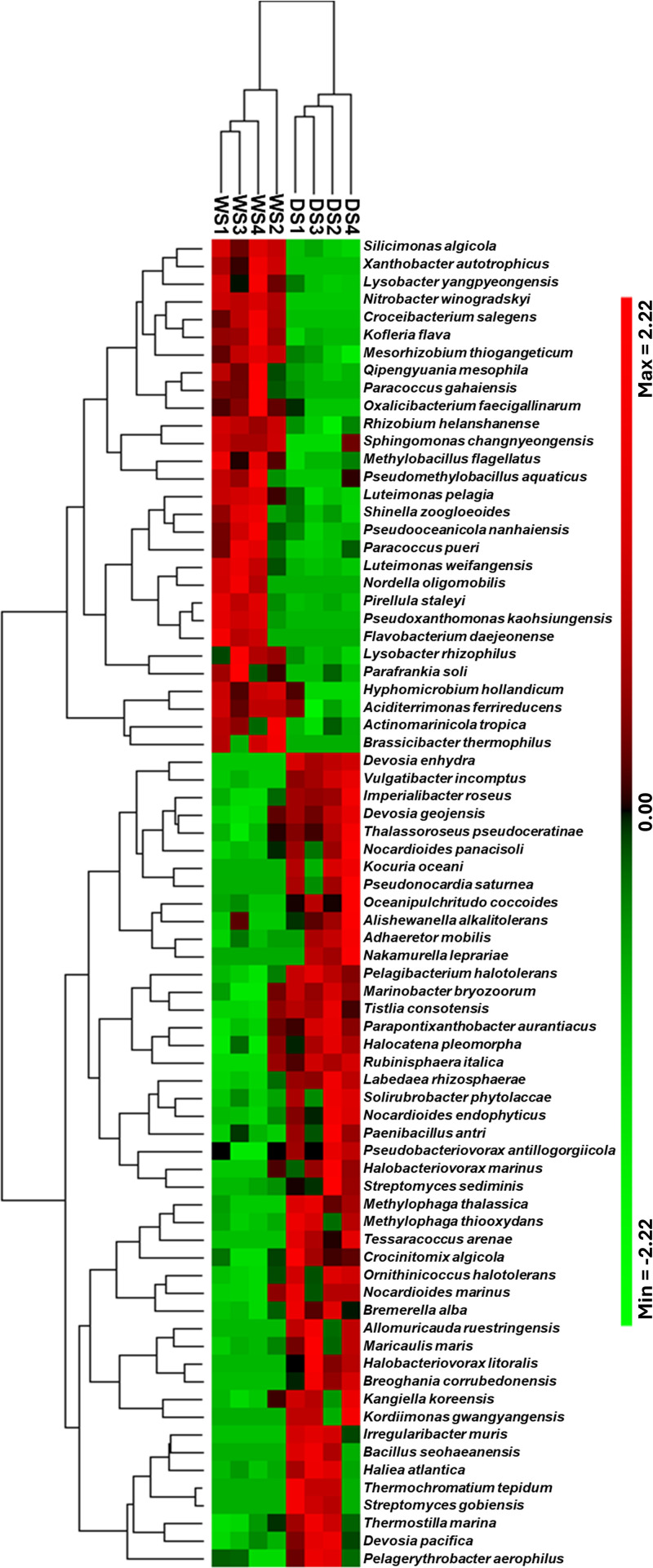
Hierarchical heatmap clustering analysis of the 75 epiphytic rhizobacterial species that showed differential accumulation at significant values (*P*≤  0.05) in response to drought. The analysis included four replicates of epiphytic rhizobacterial communities of cowpeas grown under control (WS1-WS4) and drought (DS1-DS4) conditions.

The hierarchical heatmap clustering analysis was conducted to visually compare the abundance of species and reveal community structure patterns in epiphytic bacterial communities from cowpeas under control and drought conditions. It was also used to identify clusters within bacterial abundance profiles and among treatment replicates. The heatmap analysis of the abundance profiles of the accumulated 75 species revealed two distinct clusters, which include 46 species that exhibited higher abundance in response to drought treatment, while the remaining 29 species predominantly accumulated under the control condition (Fig 5). The heatmap analysis showed that the four replicates for each treatment exhibited tight clustering by the constructed replicate dendrogram, reflecting their ASV abundance profiles similarity.

### Phylogenetic clustering reveals enrichment of drought-responsive species in specific groups

**Fig 6:**
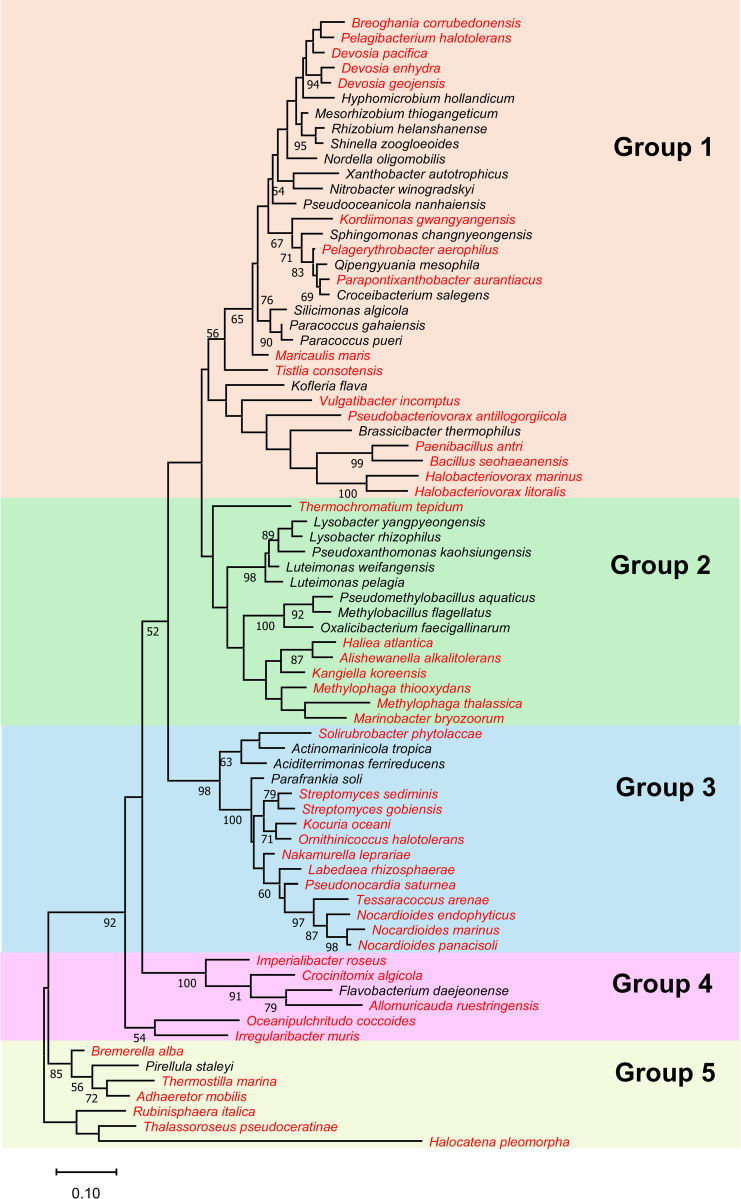
Phylogenetic analysis using the 16S rRNA gene sequences of 75 epiphytic rhizobacterial species demonstrated significant (*P*≤  0.05) differential accumulation in response to drought. Species marked in red showed a higher abundance under drought treatment. Bootstrap values greater than 50% are displayed on the phylogenetic tree.

Phylogenetic analysis of the 16S rRNA gene sequences of the differentially accumulated ASVs with a significant value (*P* ≤  0.05) was conducted to determine the evolutionary relationships among microbial species, assess community structure, and identify distinct groups within the epiphytic bacterial communities associated with cowpeas grown under control and drought treatments. Additionally, ASVs with a relatively higher abundance in response to drought were labeled on the tree to study their relationship with drought and to understand their potential role in bacterial community dynamics under drought conditions. The phylogenetic analysis identified five distinct sequence groups (Group I - Group 5), including three species-rich clades (Group 1 to Group 3) and two species-deficient clades (Group 4 and Group 5) (Fig 6). Drought-responsive species with significantly (*P* ≤  0.05) higher abundance than control were clustered within specific groups. For instance, Group 3 contained 12 out of 15 species, Group 4 included 5 out of 6 species, and Group 6 comprised 6 out of 7. On the other hand, species that exhibited significant (*P* ≤  0.05) abundance under the control treatment were remarkably enriched within clades of Group 1 and Group 2.

## Discussion

Drought stress significantly limits plant growth, even in drought-tolerant species like cowpeas. This observation was evident from the reduction in the plant height, shoot fresh and dry weight, and overall fresh weight in cowpeas. These findings align with earlier studies showing that water scarcity decreases cowpea biomass [27]. Furthermore, the observed reduction in root fresh weight under drought stress can be attributed to limited water availability, which restricts root cell expansion due to decreased turgor pressure [28]. Nevertheless, rhizospheric bacteria may contribute to certain levels of drought tolerance in cowpeas. This study shows how drought stress influences rhizospheric bacterial communities in cowpeas, especially in the alkaline soil of low organic carbon percentages typical to arid and semiarid regions, which emphasizes their role in enhancing plant resilience. The study also identifies specific bacterial species and phylogenetic clusters potentially associated with drought tolerance by analyzing microbial diversity and composition changes under drought conditions.

Cowpeas’ drought-treated epiphytic bacterial communities exhibited higher biodiversity, as reflected by increased ASV richness and Shannon index values, indicating greater microbial diversity and a more balanced species distribution than control conditions. While the Gini-Simpson index revealed consistent species evenness across groups, the elevated PD whole tree score under drought suggests greater evolutionary diversity, highlighting drought’s role as an environmental filter in selecting species from distinct evolutionary lineages. This notion suggests more resilient bacterial communities with adaptive strategies for water scarcity in the cowpea rhizosphere [29], while stable species evenness helps maintain ecosystem functions despite environmental stress [30]. The beta diversity analysis revealed that bacterial communities from plants grown under control conditions were more conserved than those from plants subjected to drought conditions. The reduced clustering of bacterial community profiles under drought conditions reflects increased diversity. The enhanced diversity can be attributed to environmental heterogeneity and species turnover, which, along with stochastic processes such as random colonization and community assembly dynamics, contribute to a more variable and less predictable community structure than the more stable and uniform profiles observed under control conditions [29]. Increasing microbial diversity in cowpeas by introducing unique taxa and fostering evolutionary diversity enhances resilience by improving ecosystem stability and adaptability through nutrient cycling, stress mitigation, and plant-microbe interactions [31,32]. At the same time, variability in rhizobacteria allows functional groups to respond to drought stress, contributing to soil health and cowpea growth [33].

Drought has exhibited variable impacts on the diversity of rhizospheric bacteria across different plant species, and this diversity sometimes depends on the strength of the applied drought, soil conditions, and the host plant species [34]. For instance, while drought enhanced the microbial diversity in the rhizosphere of cowpeas in the current study, prolonged drought treatment reduced the microbial diversity in other plant species, such as *Populus* trees [35]. In rice, drought conditions increased the complexity of the co-occurrence network in the rhizosphere. Although species richness remained unchanged, bacterial diversity increased, as measured by the Shannon index, was enhanced [36]. In the rhizosphere of cotton, drought stress increased bacterial alpha diversity, accompanied by the enrichment of specific bacterial phyla, such as Chloroflexi and Gemmatimonadetes [37]. Conversely, drought stress in sugarcane reduced bacterial diversity in the rhizosphere. This effect was more pronounced in drought-sensitive cultivars than drought-tolerant ones, the latter maintaining a stable microbiome with selective enrichment of drought-resistant taxa [38]. Similarly, in the Arabidopsis rhizosphere, analysis of α- and β-diversity revealed that drought stress did not significantly affect overall species diversity between groups but also influenced species composition [17].

*Nocardioides* was identified as the most abundant genus in this study. This Gram-positive, mesophilic, and aerobic bacterial genus, belonging to the Nocardioidaceae family, includes several plant-associated bacterial species [39]. Members of this genus have been previously cultured from plants exposed to abiotic stresses and from desert and dry environments [40]. For instance*, Nocardioides caricicola* sp. nov., an endophytic species, was isolated from the halophytic plant *Carex scabrifolia Steud* [41]. Additionally, species of *Nocardioides* have been utilized as biomarkers in the endosphere and rhizosphere of spring wheat under varying levels of drought stress [42]. Similarly, their presence has been associated with drought-stressed rhizospheres and endospheres of rice [36].

Differential accumulation analysis of the epiphytic bacteria revealed a group of species accumulated significantly in the cowpeas rhizosphere during drought treatment. These species can be prospectively cultured and tested for plant growth-promoting activities under drought conditions. The group list includes previously isolated bacterial species in drought conditions, such as *Methylophaga* spp.*,* which exhibited plant growth-promoting activities by producing auxins, gibberellins, and cytokinin phytohormones and 1-aminocyclopropane-1-carboxylic acid (ACC) deaminase, siderophore, ammonia production, and secondary metabolites [43], *Nocardioides* spp*.*, which are drought-tolerant species of desert habitats [40], *and Pseudonocardia saturnea*, which was previously identified in the rhizospheric soil of an endemic drought-tolerant grass (*Lasiurus sindicus*) [44], *Ornithinicoccus halotolerans,* previously identified in Haplocambids desert soil of UAE [45], and the desert soil of Xinjiang, north-west China [46].

The soil used in this experiment was collected from an area near the seacoast, where salinity is a significant factor. Drought and salinity stress share overlapping physiological, biochemical, and ecological responses in the rhizosphere, such as altering osmotic balance, inducing oxidative stress, and triggering the release of specific plant exudates that influence microbial community structures [ [47,48]. Consistent with these shared stress responses, the differentially accumulated bacterial list included species previously associated with saline environments. Examples include *Adhaeretor mobilis*, isolated from a halotolerant area in Germany [49], *Halobacteriovorax* spp., salt-adapted bacteria [50,51], *Haliea atlantica*, associated with the halophytic plant *Lycium ruthenicum* [52], and *Halocatena pleomorpha*, a highly halophilic archaeon isolated from saltpan soil [53], and widespread in hypersaline environments [54].

Other species included *Kangiella koreensis,* isolated from tidal flat sediments at Daepo Beach, Yellow Sea, Korea [55], *Marinobacter bryozoorum*, a halophilic marine strain [56]; and *Maricaulis maris*, a halophilic species isolated from seawater [57]. The genus Marinobacter, notably abundant in date palms under saline stress [58], further supports this connection. Additional species included *Pelagibacterium halotolerans*, a marine halotolerant bacterium isolated from the East China Sea [59], and *Tistlia consotensis,* another halotolerant bacterium [60]*. Thermochromatium tepidum*, a thermophilic purple sulfur photosynthetic bacterium found initially in Yellowstone National Park hot springs [61], was also identified with its carotenoid production [62]. The presence of these bacterial species, known for their associations with saline and other extreme environments, highlights the overlap between drought and salinity stress in forming microbial communities.

Phylogenetic analysis of the 16S rRNA genes revealed significant genetic diversification, as evidenced by species-rich clades. Interestingly, the phylogenetic analysis clustered the differentially accumulated species in response to drought into specific clades, suggesting that closely genetically related species may exhibit similar adaptive responses to drought conditions in the rhizosphere of cowpea plants. Hierarchical heat mapping analysis further supported these findings by revealing a tight clustering pattern of these species, which showed their distinct accumulation behavior under drought stress. This clustering supports the phylogenetic analysis and underlines a clear shift in bacterial diversity and composition within the rhizosphere in response to drought conditions. These findings align with previous studies indicating that drought stress selectively enriches drought-adaptive bacterial taxa, such as Actinobacteria and Proteobacteria, which are associated with plant resilience to water deficit conditions [17,63].

While 16S rRNA amplicon sequencing provides significant visions into bacterial diversity in cowpeas, it may not fully capture the functional or metabolic dynamics necessary to understand the mechanisms underlying drought tolerance. Future research should prioritize isolating key bacterial species and integrating advanced approaches such as whole genome metagenomics, metatranscriptomics, and metabolomics to comprehensively investigate these microbial communities’ functional roles and interactions in enhancing plant resilience.

## Conclusions

The study reveals that drought stress significantly reshapes the bacterial community in the rhizosphere of cowpea plants, which enhance biodiversity and promoting the accumulation of specific bacterial species that may aid in plant survival under drought. The bacterial communities included species previously identified in drought, heat, and saline conditions, supporting the validity of this analysis. These species hold strong potential for future isolation and testing for drought-promoting activities. The phylogenetic clustering of these drought-adapted species suggests that evolutionary relationships could be associated with their ability to withstand water-limited conditions. These findings highlight the potential role of the rhizospheric microbiome in plant drought tolerance, which could have applications in agricultural practices such as biofertilizer development for drought-prone environments.

## Supporting information

S1 Fig The relative taxonomic abundance of taxa units classified at phylum (A), class (B), and order (C) levels of the microbial communities identified when cowpea plants grown under control (WS1-WS4) and drought (DS1-DS4) conditions. (TIF)

S1 Table The identified ASVs and their taxonomy obtained from epiphytic rhizobacterial communities from cowpea plants grown under control conditions (WS1–WS4) and drought conditions (DS1–DS4), along with a differential analysis of their abundance across these groups. (XLSX)

S2 Table The relative abundance of phyla, classes, and orders within epiphytic rhizobacterial communities associated with cowpea plants was analyzed under control conditions (well-watered, WS) and drought stress conditions (DS). The analysis was based on the average values of biological replicates for each treatment group. Differential abundance analysis was conducted to identify variations between WS and DS groups, with statistical significance determined at *P* ≤  0.05. (XLSX)

S3 Table Species and their abundance across four replicates of epiphytic rhizobacterial communities from cowpea plants grown under control conditions (WS1–WS4) and drought conditions (DS1–DS4), along with a differential analysis of their abundance across these groups. The significant differences were calculated at *P* ≤  0.05.
(XLSX)
