# Supplementary figures and images for: Drought-induced microbial dynamics in cowpea rhizosphere: Exploring bacterial diversity and bioinoculant prospects
Source: PLoS One. 2025 Mar 25;20(3):e0320197. doi: 10.1371/journal.pone.0320197 (PMC11936235; doi:10.1371/journal.pone.0320197)

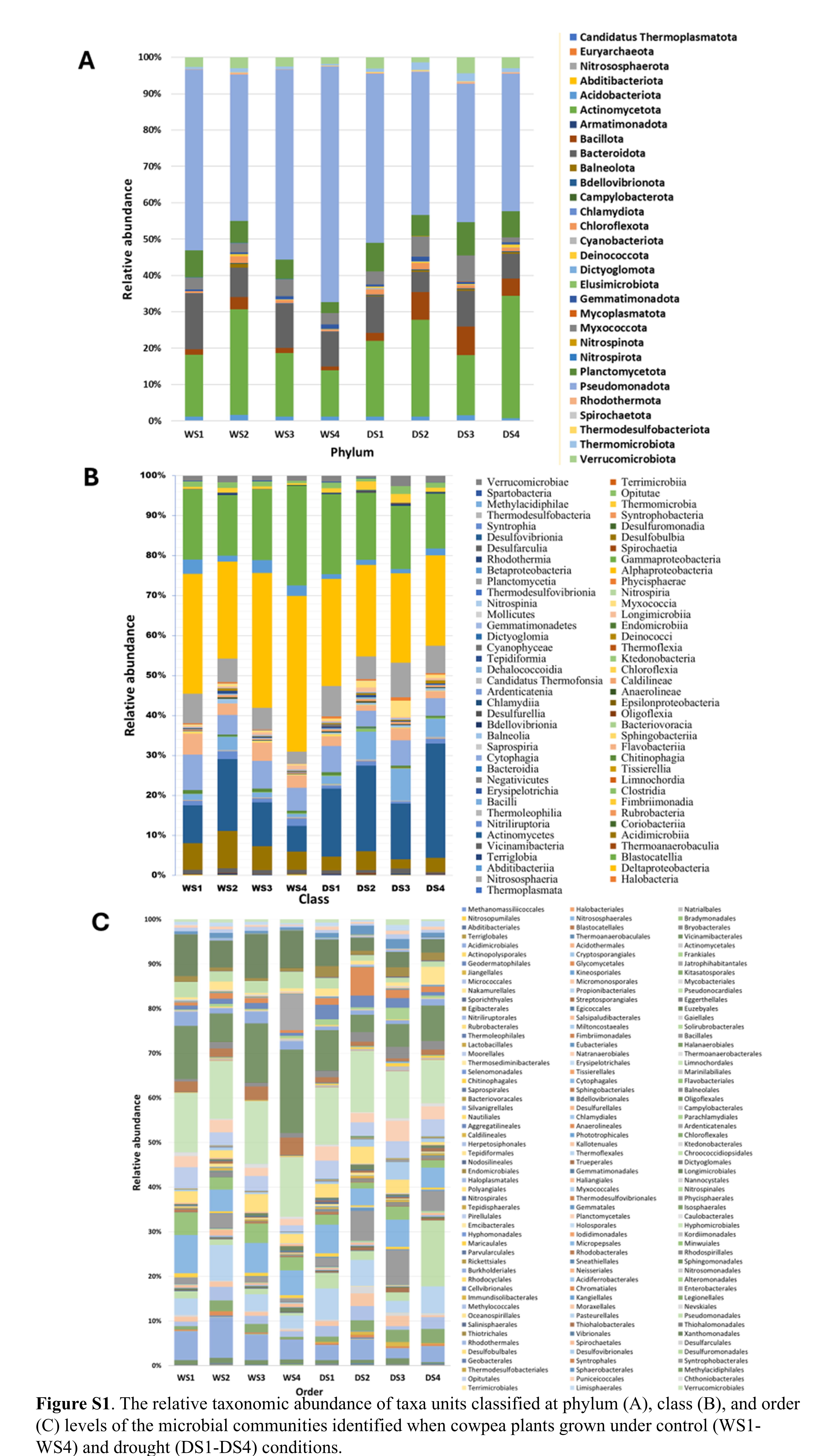

Supplement: S1 Fig — (TIF) [file pone.0320197.s001.tif]
